# Supplementary material for: Saccharomyces boulardii (CNCM I-745) improves intestinal damage in sepsis by remodeling intestinal flora structure
Source: Front Cell Infect Microbiol. 2025 Jul 31;15:1602792. doi: 10.3389/fcimb.2025.1602792 (PMC12350329; doi:10.3389/fcimb.2025.1602792)
Supplement: Supplementary file 1 [file DataSheet1.zip › summary/summary/7_Differential_analysis/1_diff_abundance/5_Family/Group.AW4_vs_AW0/Group.AW4_vs_AW0_diff_barplot.pdf]

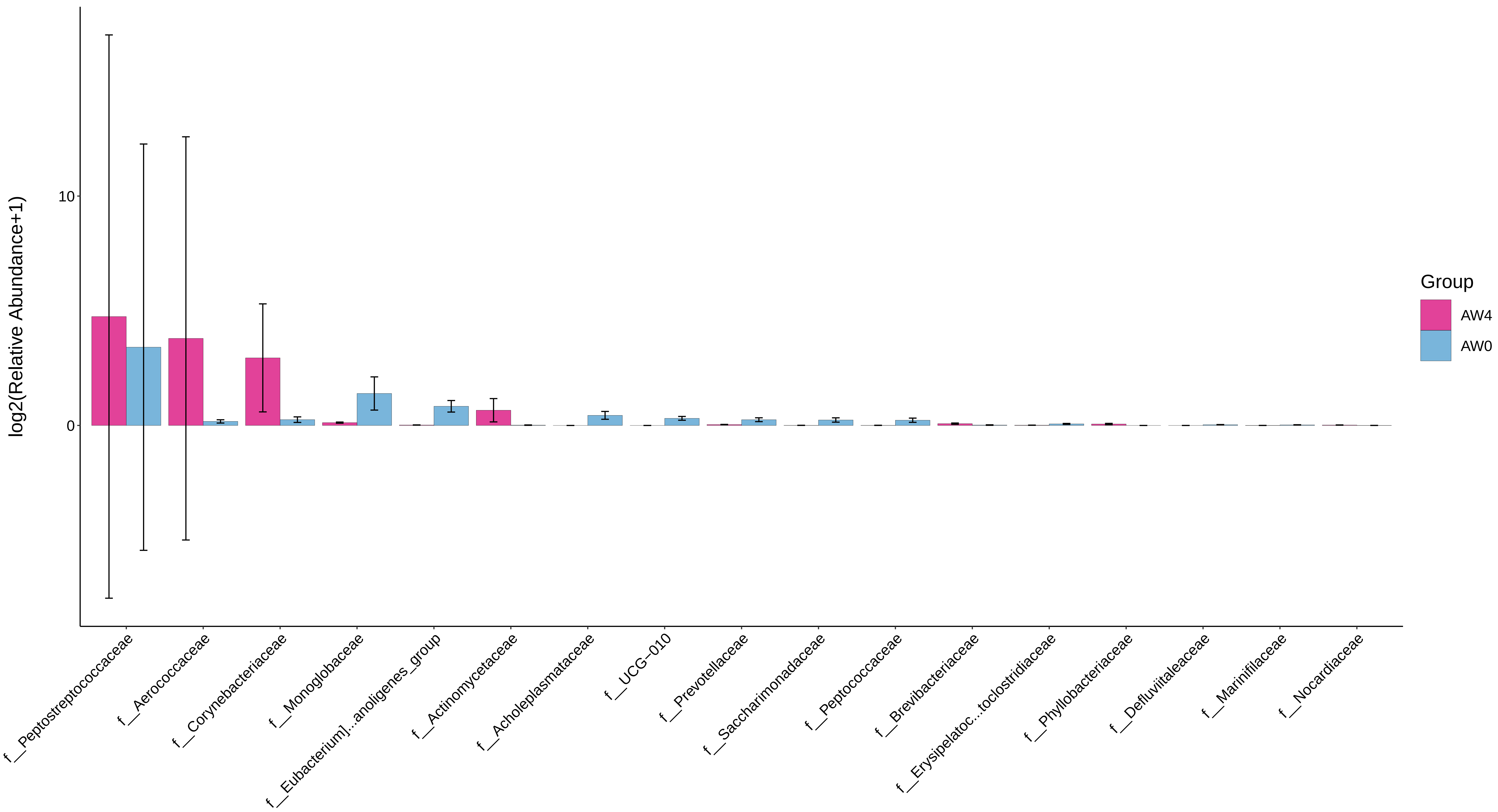

log2(Relative Abundance+1)
10
0
Group
AW4
AW0
f__Peptostreptococcaceae
f__Aerococcaceae
f__Corynebacteriaceae
f__Monoglobaceae
f__Eubacterium]...anoligenes_group
f__Actinomycetaceae
f__Acholeplasmataceae
f__UCG–010
f__Prevotellaceae
f__Saccharimonadaceae
f__Peptococcaceae
f__Brevibacteriaceae
f__Erysipelatoc...toclostridiaceae
f__Phyllobacteriaceae
f__Defluviitaleaceae
f__Marinifilaceae
f__Nocardiaceae
